# Effectiveness evaluation of adjuvant concurrent chemoradiotherapy for patients with positron emission tomography‐staged esophageal squamous cell carcinoma after complete resection: A population‐based cohort study

**DOI:** 10.1111/1759-7714.14476

**Published:** 2022-06-03

**Authors:** Hsin‐Yuan Fang, Yu‐Sen Lin, Chien‐Kuang Chen, Jian‐Xun Chen, Ting‐Yu Lu, Tzu‐Min Huang, Te‐Chun Hsieh, Yu‐Cheng Kuo, Chen‐Yuan Lin, Ming‐Yu Lien, Chi‐Ching Chen, Chia‐Chin Li, Chun‐Ru Chien

**Affiliations:** ^1^ Department of Chest Surgery China Medical University Hospital Taichung Taiwan; ^2^ School of Medicine, College of Medicine China Medical University Taichung Taiwan; ^3^ Department of Nuclear Medicine and PET Center China Medical University Hospital Taichung Taiwan; ^4^ Department of Biomedical Imaging and Radiological Science China Medical University Taichung Taiwan; ^5^ Department of Radiation Oncology China Medical University Hsinchu Hospital Hsinchu Taiwan; ^6^ Division of Hematology and Oncology, Department of Internal Medicine China Medical University Hospital Taichung Taiwan; ^7^ Department of Radiation Oncology China Medical University Hospital Taichung Taiwan; ^8^ Department of Medical Research China Medical University Hsinchu Hospital Hsinchu Taiwan

**Keywords:** adjuvant concurrent chemoradiotherapy, esophageal squamous cell carcinoma, esophagectomy, positron emission tomography staging

## Abstract

**Background:**

The role of adjuvant concurrent chemoradiotherapy (ACCRT) is unclear for patients with esophageal squamous cell carcinoma (ESCC) who receive esophagectomy with clean margins. We compared the survival of the ACCRT versus observation groups for these patients staged with positron emission tomography (PET) via a population‐based approach.

**Methods:**

Eligible patients with locally advanced ESCC diagnosed between 2011 and 2017 were identified via the Taiwan Cancer Registry. We used propensity score (PS) weighting to balance observable potential confounders between groups. The hazard ratios (HR) of death and incidence of esophageal cancer mortality (IECM) were compared between the ACCRT and observation groups. We also evaluated overall survival (OS) in subgroups of either with or without lymph node metastases.

**Results:**

Our primary analysis consisted of 105 patients in whom the covariates were well balanced after PS weighting. The HR for death when ACCRT was compared with observation was 0.58 (95% confidence interval 0.28–1.21, *p* = 0.15). The results were also not significantly different for IECM or in the subgroup analyses.

**Conclusion:**

We found that for patients with PET‐staged ESCC who received esophagectomy with clean margins, the survival was not statistically different between ACCRT and observation. Further studies (randomized or larger sample size) are needed to clarify this issue.

## Graphical abstract

We observed a trend in favor of ACCRT group (vs. observation group) for patients with PET‐staged ESCC who received esophagectomy with clean margins in this population‐based non‐randomized study.

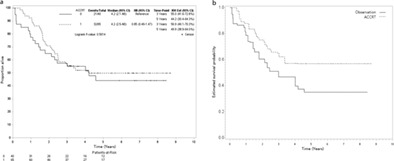

## INTRODUCTION

Esophageal cancer is one of the leading causes of cancer mortality around the world, including Taiwan.^1^ The most common histology is adenocarcinoma in Western countries, whereas it is squamous cell carcinoma in Asian countries.^1^, ^2^

For patients with esophageal squamous cell carcinoma (ESCC) who receive esophagectomy with involved margins, adjuvant concurrent chemoradiotherapy (ACCRT) is commonly suggested in the treatment guidelines.^3^ However, the optimal adjuvant treatment for those with clear margins is less straightforward. Although ACCRT is not favored in the North American guidelines,^3^, ^4^ there was only one relevant randomized controlled trial (RCT) according to a systematic review published in 2020.^5^ This RCT was published a decade ago in Asia and showed favorable outcomes for those treated with ACCRT.^6^ However, this study^6^ involved staging by computed tomography (CT), whereas the use of positron emission tomography (PET) rather than CT‐only has been strongly favored in the modern era.^3^, ^7^ When we searched PubMed further using the keywords “(esophageal cancer) AND (squamous cell carcinoma) AND (adjuvant) OR (post operative) OR (postoperative) AND (concurrent chemoradiotherapy) AND (positron emission tomography)” in January 2022, we could not find relevant studies.

Because of the above‐mentioned paucity of data regarding ACCRT, especially in the modern era, we compared the survival of ACCRT versus observation for patients with PET‐staged ESCC treated via esophagectomy with clear margins based on the modern cancer registry data from Taiwan.

## MATERIAL AND METHODS

### Data source

In this retrospective cohort study based on registry data, the analyzed data with personal identifiers removed were obtained from the Health and Welfare Data Science Center (HWDC) database, which included the Taiwan Cancer Registry (TCR), death registration, and reimbursement data for the entire Taiwan population provided by the Bureau of National Health Insurance (NHI). The TCR is a high‐quality database^8^ that provides comprehensive information such as patient demographics and patient and disease characteristics. This study was approved by the Central Regional Research Ethics Committee at China Medical University Taichung Taiwan (CRREC‐108‐080 [CR2]).

### Study population and intervention

The inclusion criteria of our study population were: (a) patients with thoracic ESCC diagnosed within 2011–2017 from the TCR via the International Classification of Disease for Oncology 3rd edition (ICD‐O‐3) reference and histology codes; (b) had PET for staging; (c) received upfront esophagectomies with clear margins; (d) had locally advanced disease as pathological stage T3‐4N0M0 or pT1‐4 N1‐3 M0 as defined by the 7th American Joint Committee on Cancer (AJCC); and (e) were 18–70 years old. The exclusion criteria were: (a) those with multiple treatment records in the TCR; and (b) those with prior cancer(s). These inclusion/exclusion criteria were modified from our clinical and research experience as well as previous studies.^6^, ^9^, ^10^

Regarding intervention, we identified patients treated with either ACCRT (ACCRT group) using external beam radiotherapy 45–50.4 Gy in conventional fractionation according to the records in the TCR or no further systemic or radiotherapy (observation group).

### Covariates

We included the following covariates as modified from recent relevant studies and our clinical and research experience.^6^, ^9^, ^10^ Patient demographics (age, gender, and residency); patient characteristics (comorbidity, body mass index [BMI], drinking, and smoking); and disease characteristics (tumor size, tumor grade, tumor location, T‐stage, and number of lymph node metastases as well as p‐stage) were defined as follows. The patient residency region was classified as “northern Taiwan” or “non‐north.” Comorbidity was determined by the modified Charlson comorbidity index score^11^ and classified as “with” or “without.” The smoking and drinking were classified as “yes” or “no.” The p‐stage was classified as “2” versus “3.” The pathological T‐stage was classified as “1–2” or “3–4.” Grade was classified as “well/moderately differentiated” or “poorly/undifferentiated.” Tumor location was classified as “upper,” “middle,” or lower.

### Analyses

The primary outcome of interest was overall survival (OS). We also evaluated the impact of intervention (ACCRT vs. observation) on incidence of esophageal cancer mortality (IECM). These endpoints were determined based on records in the TCR and death registry (censored on December 31, 2019). We adopted a propensity score (PS) approach and used propensity score weighting (PSW) as the framework for primary analyses as advocated in previous studies.^12^, ^13^, ^14^, ^15^ We estimated the probability of receiving ACCRT (vs. observation) with a logistic regression model based on all the above covariates, and then assessed the balance of covariates between groups after PSW using overlap weight^16^ via the standardized difference (SDif).^12^ We compared the hazard ratio (HR) of death between the ACCRT and observation groups during the entire follow‐up period via Cox proportional hazards model in the weighted sample for point estimation and used the bootstrap method to estimate the 95% confidence interval (95% CI).^17^, ^18^, ^19^ We used an E‐value to assess the robustness of our finding regarding potential unmeasured confounder(s), as suggested in the literature^20^, ^21^ because the PS approach can only be valid under the assumption of no unmeasured confounder(s). We took a competing risk approach to compare IECM between groups.^22^ We performed the following supplementary analyses (SA) for subgroup analyses: SA‐1 for patients with pathological lymph node metastases (pN+) and SA‐2 for those without (pN0), whereas SA‐3 for patients with pathological stage II (p‐stage 2) and SA‐4 for patients with pathological stage III (p‐stage 3) because the role of adjuvant thoracic radiotherapy may vary according to lymph node status or pathological stage.^23^ We used SAS v.9.4 software (SAS Institute) for statistical analyses.

## RESULTS

### Study population

**FIGURE 1:**
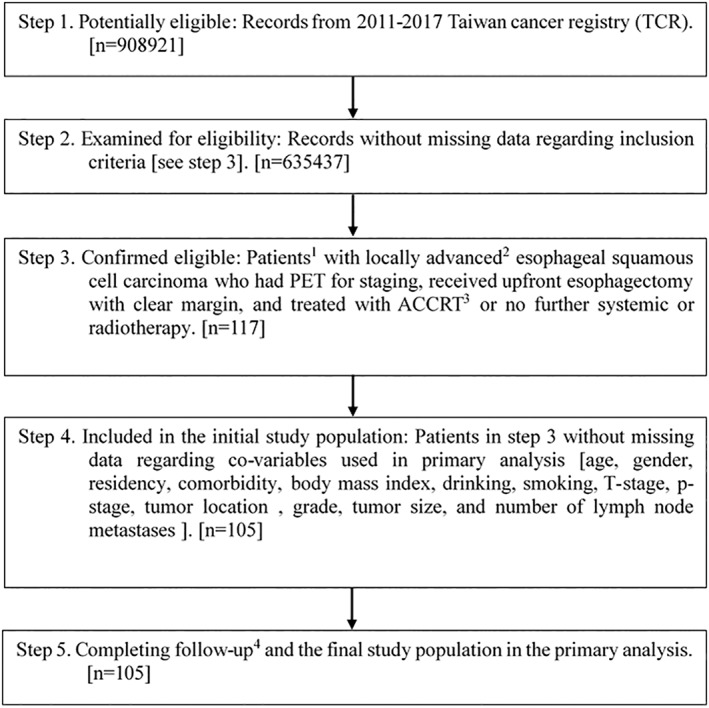
STROBE study flowchart and the number of individuals at each stage of the study. (a) We only included those treated (class 1–2) with only one record to ensure data consistency, age 18–70 years old. (b) The Seventh AJCC staging pathological stage T3‐4N0M0 or pT1‐4 N1‐3 M0. (c) Using external beam radiotherapy 45–50. 4 Gy in conventional fractionation. (d) Without missing information in the TCR and death registry.

We identified 105 patients (65 for ACCRT group and 40 for observation group) as our primary study population as shown in Figure 1.^24^ We achieved covariate balance after PSW, although some imbalance was seen before PSW as shown in Table 1.

**TABLE 1:** Patient characteristics of the study population in the primary analysis

	Patient characteristics before PSW			Patient characteristics (%) after PSW^a^		
	ACCRT (*n* = 65)	Observation (*n* = 40)	Standardized difference^b^	ACCRT	Observation	Standardized difference^b^
	No. (%)^b^ or mean (SD)^b^	No. (%)^b^ or mean (SD)^b^				
Age (y)	52.35 (8.73)	57.73 (7.24)	0.670	55.99	55.99	≈0
Gender						
Female	^d^	^d^	0.165	^d^	^d^	≈0
Male	^d^	^d^		^d^	^d^	
Residency						
Non‐north	37 (57)	21 (53)	0.089	57	57	≈0
North	28 (52)	19 (48)		43	43	
Comorbidity						
Without	58 (89)	35 (87)	0.054	92	92	≈0
With^c^	7 (11)	5 (13)		8	8	
BMI (kg/m^2^)	22.20 (2.74)	22.46 (3.20)	0.087	22.01	22.01	≈0
Drinking						
No	5 (8)	8 (20)	0.362	12	12	≈0
Yes	60 (92)	32 (80)		88	88	
Smoking						
No	6 (9)	4 (10)	0.026	9	9	≈0
Yes	59 (91)	36 (90)		91	91	
Grade						
Poorly	20 (31)	5 (13)	0.455	18	18	≈0
Well/moderately differentiated	45 (69)	35 (87)		82	82	
T‐stage						
1–2	20 (31)	12 (30)	0.017	34	34	≈0
3–4	45 (69)	28 (70)		66	66	
P‐stage						
2	28 (43)	30 (75)	0.686	66	66	≈0
3	37 (57)	10 (25)		34	34	
Tumor location						
Upper	^d^	^d^		^d^	^d^	
Middle	^d^	^d^	0.096	^d^	^d^	≈0
Lower	^d^	^d^	0.166	^d^	^d^	≈0
Tumor size (mm)	41.06 (18.41)	40.03 (18.40)	0.056	40.65	40.65	≈0
No. of lymph node metastases	2.02 (2.36)	1.05 (1.84)	0.457	1.44	1.44	≈0

Abbreviations: ACCRT, adjuvant concurrent chemoradiotherapy; BMI, body mass index; PSW, propensity‐score weighting; SD, standard deviation.

^a^ Weighted mean or proportion for each group (rounded).

^b^ Rounded.

^c^ Modified Carlson comorbidity score ≥1.

^d^ The exact numbers were not reported because of a Health and Welfare Data Science Center (HWDC) database center policy to avoid numbers in single cells (≤2).

### Primary analyses

**FIGURE 2:**
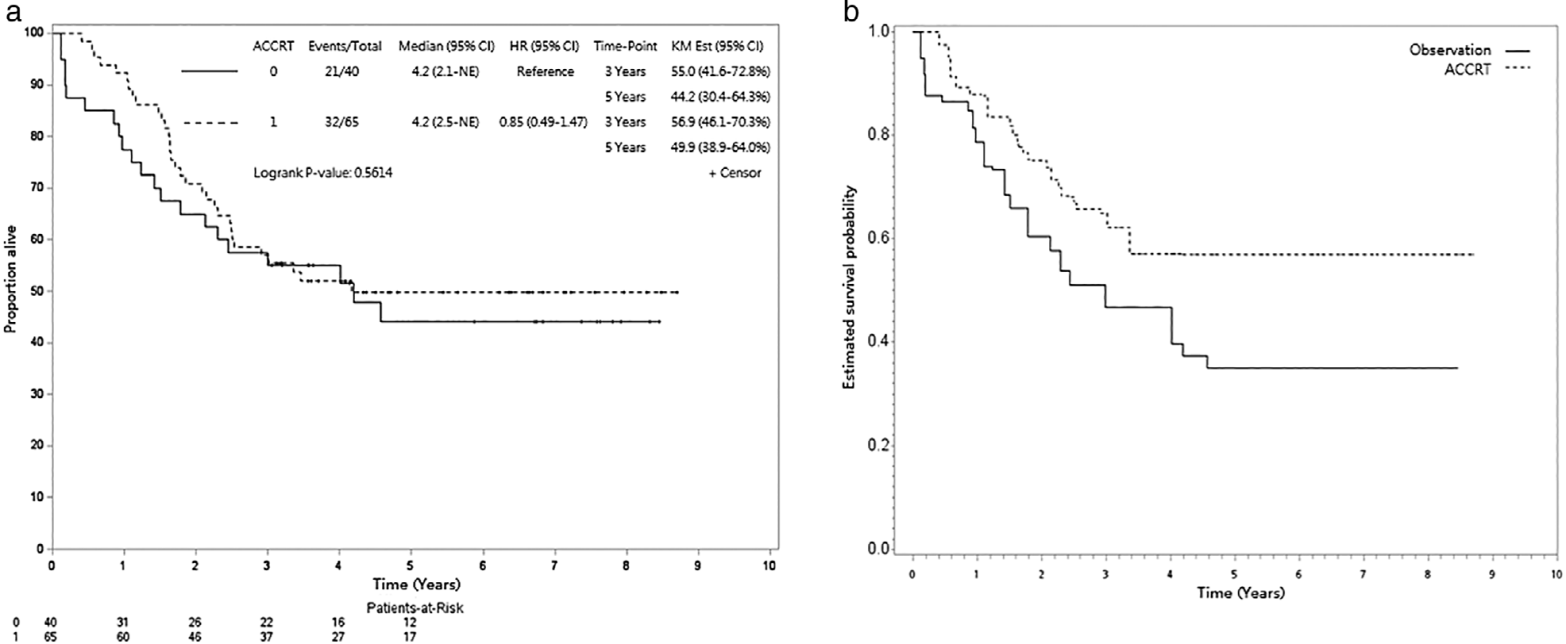
(a) Kaplan–Meier unadjusted overall survival curve (in years), and (b) the overlap weight‐adjusted overall survival curve (in years) in the primary analysis.

After a median follow‐up of 40 months (range, 1–104 months), 53 deaths were observed (32 and 21 patients for ACCRT and observation groups, respectively). The median follow‐up was 72 months (range, 37–104) for survivors. The 5‐year OS rates were 50% and 44% for the ACCRT and observation groups, respectively, in the unadjusted analysis (log‐rank test, *p* = 0.56) (Figure 2(a)). The overlap weights adjusted OS curve is shown in Figure 2(b). The 3–5‐year OS rates for both groups were 65%–57% (ACCRT group) and 47%–35% (observation group), respectively. When the ACCRT group was compared to the observation group, the HR of death was 0.58 (95% CI, 0.28–1.21, *p* = 0.15). The observed HR of 0.58 for OS could be explained by an unmeasured confounder that was associated with both selections of treatment (ACCRT vs. observation) and outcome (live vs. death) by a risk ratio of 2.27 (E‐value)‐fold each, but weaker confounding could not do so.^20^ The HR for IECM was 0.84 (95% CI, 0.31–2.29, *p* = 0.73).

### Supplementary analyses

**FIGURE 3:**
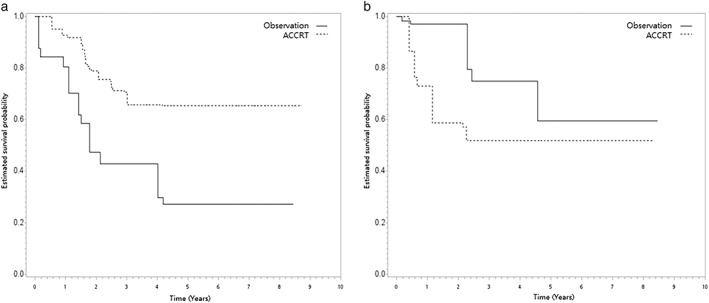
(a) The overlap weight‐adjusted overall survival curve (in years) in the SA‐1 (pN+), and (b) the overlap weight‐adjusted overall survival curve (in years) in the SA‐2 (pN0).

In both SA‐1 and SA‐2, we achieved covariate balance after PSW although some imbalance was seen before PSW as shown in Tables 2 and 3. When the ACCRT group was compared to the observation group, the HR of death was 0.38 (95% CI, 0.04–3.44, *p* = 0.39) for SA‐1 (pN+) and 1.79 (95% CI, 0.07–44.81, *p* = 0.36) for SA‐2 (pN0), respectively. The overlap weights adjusted OS curves are shown in Figure 3(a),(b). In both SA‐3 (p‐stage 2) and SA‐4 (p‐stage 3), we also achieved covariate balance after PSW although some imbalance was seen before PSW (see Tables S1 and S2). There was also no statistical difference in PSW‐adjusted OS for p‐stage 2 (*p* = 0.4) or p‐stage 3 (*p* = 0.39).

**TABLE 2:** Patient characteristics of the study population in the SA‐1 (pN+)

	Patient characteristics before PSW			Patient characteristics (%) after PSW^a^		
	ACCRT (*n* = 53)	Observation (*n* = 19)	Standardized difference^b^	ACCRT	Observation	Standardized difference^b^
	No. (%)^b^ or mean (SD)^b^	No. (%)^b^ or mean (SD)^b^				
Age (y)	52.79 (9.27)	58.79 (8.25)	0.684	57.15	57.15	≈0
Gender						
Female	^d^	^d^	0.456	^d^	^d^	≈0
Male	^d^	^d^		^d^	^d^	
Residency						
Non‐north	32 (60)	9 (47)	0.263	63	63	≈0
North	21 (40)	10 (53)		37	37	
Comorbidity						
Without	48 (91)	16 (84)	0.192	88	88	≈0
With^c^	5 (9)	3 (16)		12	12	
BMI (kg/m^2^)	22.37 (2.64)	22.67 (3.09)	0.106	22.06	22.06	≈0
Drinking						
No	^d^	^d^	0.404	^d^	^d^	≈0
Yes	^d^	^d^		^d^	^d^	
Smoking						
No	^d^	^d^	0.036	^d^	^d^	≈0
Yes	^d^	^d^		^d^	^d^	
Grade						
Poorly	^d^	^d^	0.461	^d^	^d^	≈0
Well/moderately differentiated	^d^	^d^		^d^	^d^	
T‐stage						
1–2	20 (38)	12 (63)	0.526	51	51	≈0
3–4	33 (62)	7 (37)		49	49	
P‐stage						
2	16 (30)	10 (53)	0.468	48	48	≈0
3	37 (70)	9 (47)		52	52	
Tumor location						
Upper	^d^	^d^		^d^	^d^	
Middle	^d^	^d^	0.441	^d^	^d^	≈0
Lower	^d^	^d^	0.563	^d^	^d^	≈0
Tumor size (mm)	41.00 (19.05)	37.79 (17.34)	0.176	41.17	41.17	≈0
No. of lymph node metastases	2.47 (2.38)	2.21 (2.15)	0.115	2.39	2.39	≈0

Abbreviations: ACCRT, adjuvant concurrent chemoradiotherapy; BMI, body mass index; PSW, propensity‐score weighting; SD, standard deviation.

^a^ Weighted mean or proportion for each group (rounded).

^b^ Rounded.

^c^ Modified Carlson comorbidity score ≥1.

^d^ The exact numbers were not reported because of a Health and Welfare Data Science Center (HWDC) database center policy to avoid numbers in single cells (≤2).

**TABLE 3:** Patient characteristics of the study population in the SA‐2 (pN0)

	Patient characteristics before PSW			Patient characteristics (%) after PSW^a^		
	ACCRT (*n* = 12)	Observation (*n* = 21)		ACCRT	Observation	Standardized difference^b^
	No. (%)^b^ or mean (SD)^b^	No. (%)^b^ or mean (SD)^b^	Standardized difference^b^			
Age (y)	50.42 (5.71)	56.76 (6.23)	1.062	52.85	52.85	≈0
Gender						
Female	^d^	^d^	0.042	^d^	^d^	≈0
Male	^d^	^d^		^d^	^d^	
Residency						
Non‐north	5 (42)	12 (57)	0.313	57	57	≈0
North	7 (58)	9 (43)		43	43	
Comorbidity						
Without	^d^	^d^	0.213	^d^	^d^	≈0
With^c^	^d^	^d^		^d^	^d^	
BMI (kg/m^2^)	21.47 (3.20)	22.27 (3.36)	0.244	22.21	22.21	≈0
Drinking						
No	^d^	^d^	0.753	^d^	^d^	≈0
Yes	^d^	^d^		^d^	^d^	
Smoking						
No	^d^	^d^	0.042	^d^	^d^	≈0
Yes	^d^	^d^		^d^	^d^	
Grade						
Poorly	5 (42)	3 (14)	0.640	22	22	≈0
Well/moderately differentiated	7 (58)	18 (86)		78	78	
T‐stage						
1–2	^d^	^d^		^d^	^d^	≈0
3–4	^d^	^d^		^d^	^d^	
P‐stage						
2	^d^	^d^	0.316	^d^	^d^	≈0
3	^d^	^d^		^d^	^d^	
Tumor location						
Upper	^d^	^d^		^d^	^d^	
Middle	^d^	^d^	0.242	^d^	^d^	≈0
Lower	^d^	^d^	0.313	^d^	^d^	≈0
Tumor size (mm)	41.33 (16.00)	42.05 (19.50)	0.040	35.83	35.83	≈0

Abbreviations: ACCRT, adjuvant concurrent chemoradiotherapy; BMI, body mass index; PSW, propensity‐score weighting; SD, standard deviation.

^a^ Weighted mean or proportion for each group (rounded).

^b^ Rounded.

^c^ Modified Carlson comorbidity score ≥1.

^d^ The exact numbers were not reported because of a Health and Welfare Data Science Center (HWDC) database center policy to avoid numbers in single cells (≤2).

## DISCUSSION

In this population‐based study, we found that for patients with PET staged ESCC who received esophagectomy with clear margins, the survival was not statistically different between adjuvant concurrent chemoradiotherapy and observation. To the best of our knowledge, our study was first to address this topic.

The numeric trend found in our study (in favor of ACCRT) was compatible with previous RCT or observational studies without mandatory PET. Lv et al.^6^ reported 3‐year OS of 63% for ACCRT versus 51% for observation, whereas 65% versus 47% was observed in our study after PSW adjustment. In another recently published RCT (also limited by lack of mandatory PET), Ni et al.^25^ reported 3‐year OS of 66.5% for ACCRT versus 48% for observation (*p* = 0.016). Hsu et al.^26^ reported the HR of death to be 0.63 when ACCRT was compared to observation, whereas we found the HR of death to be 0.58 in our study. However, statistical significance was not reached in our study. We summarized our finding with the available RCTs in the Table 4. Therefore, our finding (no statistically significant difference between ACCRT vs. observation) was also compatible with current guidelines,^3^, ^4^ which do not recommend routine ACCRT for these patients.

**TABLE 4:** Comparison of our finding with the available RCTs

Studies	Patient characteristics	Interventions	Outcomes
Lv et al.^6^	Age mode (y): 60–70 Male (%)^b^: 63 Staging: by CT PET: not mentioned	OBS vs. NCCRT vs. ACCRT	3‐y OS (%)^b^: 51 vs. 63 vs. 63
Ni et al.^25^	Age median (y): 59 Male (%)^b^: 90 Staging: by CT PET: not routine but only if needed (details not reported)	OBS vs. ART vs. ACCRT	3‐y OS (%)^b^: 48 vs. 61 vs. 67
Current study	Age mean (y)^a^ ^,^ ^b^: 56 Male: predominant^c^ Staging: PET required	OBS vs. ACCRT	3‐y OS (%)^a^ ^,^ ^b^: 47 vs. 65

Abbreviations: ACCRT, adjuvant concurrent chemoradiotherapy; ART, adjuvant radiotherapy; CT, computed tomography; NCCRT, neoadjuvant concurrent chemoradiotherapy; OBS, observation; OS, overall survival; PET, positron emission tomography; RCT, randomized controlled trial.

^a^ After propensity score weighting.

^b^ Rounded.

^c^ The exact numbers were not reported because of a Health and Welfare Data Science Center (HWDC) database center policy to avoid numbers in single cells (≤2).

Further studies, such as RCT or studies with larger sample sizes are needed to clarify this issue. However, when we searched the clinical trial registry (https://clinicaltrials.gov/) using keywords “Esophageal Squamous Cell Carcinoma | esophagectomy concurrent chemoradiotherapy | positron emission tomography,” we did not find any relevant RCT.

In addition to the limitation of a moderate study sample size as mentioned above, there were several other limitations in our study. First, the nonrandomized nature of our study made potential unmeasured confounder(s) (such as systemic therapy or radiotherapy details or micro‐metastases status)^27^, ^28^, ^29^ always an issue although we used a PS method to adjust for observable covariates. Therefore, we reported the E‐value to assess the robustness of our results to potential unmeasured confounder(s). Second, we did not investigate other endpoints like disease control, toxicity, or quality of life because of uncertainty in data quality or accessibility. Furthermore, the generalizability of our study may be limited by the wide‐spread use of neoadjuvant concurrent chemoradiotherapy and the increasing role of immunotherapy. For example, adjuvant immunotherapy had been reported to be beneficial for complete resected stage II or III esophageal cancer that had received neoadjuvant chemoradiotherapy and had residual pathological disease.^30^ Therefore, the role of adjuvant radiotherapy should be further evaluated in the era of immunotherapy.

## CONCLUSION

We found that for patients with PET‐staged ESCC who received esophagectomy with clear margins, the survival was not statistically different between adjuvant concurrent chemoradiotherapy and observation. Further studies such as RCT or studies with larger sample sizes are needed to clarify this issue.

## CONFLICT OF INTEREST

All authors declare no conflict of interest.

## Supporting information


**TABLE S1** Patient characteristics of the study population in the SA‐3 (p‐stage 2)
**TABLE S2** Patient characteristics of the study population in the SA‐4 (p‐stage 3)
