# Supplementary material for: Effectiveness evaluation of adjuvant concurrent chemoradiotherapy for patients with positron emission tomography‐staged esophageal squamous cell carcinoma after complete resection: A population‐based cohort study
Source: Thorac Cancer. 2022 Jun 3;13(13):1986–93. doi: 10.1111/1759-7714.14476 (PMC9250842; doi:10.1111/1759-7714.14476)
Supplement: Supplementary file 1 — TABLE S1 Patient characteristics of the study population in the SA‐3 (p‐stage 2) TABLE S2 Patient characteristics of the study population in the SA‐4 (p‐stage 3) [file TCA-13-1986-s001.docx]

Table S1. Patient characteristics of the study population in the SA-3 [p-stage 2]

|  |  | Patient characteristics before PSW | | |  | Patient characteristics (%) after PSW^a^ | | |
| --- | --- | --- | --- | --- | --- | --- | --- | --- |
|  |  | ACCRT  (n=28) | Observation  (n=30) |  |  | ACCRT | Observation |  |
|  |  | Number (%)^b^ or mean (SD)^b^ | Number (%)^b^ or mean (SD)^b^ | Standardized difference^b^ |  |  |  | Standardized difference^b^ |
| Age (years) |  | 53.14 (6.72) | 56.43 (6.55) | 0.496 |  | 54.39 | 54.39 | ≈ 0 |
| Gender | Female | ^d^ | ^d^ | 0.251 |  | ^d^ | ^d^ | ≈ 0 |
|  | Male | ^d^ | ^d^ |  |  | ^d^ | ^d^ |  |
| Residency | Non-north | 16 (57) | 15 (50) | 0.144 |  | 56 | 56 | ≈ 0 |
|  | North | 12 (43) | 15 (50) |  |  | 44 | 44 |  |
| Comorbidity | Without | 23 (82) | 26 (87) | 0.125 |  | 91 | 91 | ≈ 0 |
|  | With^c^ | 5 (18) | 4 (13) |  |  | 9 | 9 |  |
| BMI (kg/m^2^) |  | 21.81 (2.89) | 22.90 (3.26) | 0.355 |  | 22.19 | 22.19 | ≈ 0 |
| Drinking | No | ^d^ | ^d^ | 0.540 |  | ^d^ | ^d^ | ≈ 0 |
|  | Yes | ^d^ | ^d^ |  |  | ^d^ | ^d^ |  |
| Smoking | No | ^d^ | ^d^ | 0.102 |  | ^d^ | ^d^ | ≈ 0 |
|  | Yes | ^d^ | ^d^ |  |  | ^d^ | ^d^ |  |
| Grade | Poorly | 7 (25) | 5 (17) | 0.206 |  | 22 | 22 | ≈ 0 |
|  | Well/moderately differentiated | 21 (75) | 25 (83) |  |  | 78 | 78 |  |
| T-stage | 1–2 | 16 (57) | 10 (33) | 0.493 |  | 46 | 46 | ≈ 0 |
|  | 3–4 | 12 (43) | 20 (67) |  |  | 54 | 54 |  |
| Tumor location | Upper | ^d^ | ^d^ |  |  | ^d^ | ^d^ |  |
|  | Middle | ^d^ | ^d^ | 0.072 |  | ^d^ | ^d^ | ≈ 0 |
|  | Lower | ^d^ | ^d^ | 0.077 |  | ^d^ | ^d^ | ≈ 0 |
| Tumor size (mm) |  | 35.21 (19.11) | 36.73 (16.53) | 0.085 |  | 36.40 | 36.40 | ≈ 0 |
| Number of lymph node metastases |  | 0.75 (0.75) | 0.17 (0.73) | 0.382 |  | 0.60 | 0.60 | ≈ 0 |

ACCRT, adjuvant concurrent chemoradiotherapy; BMI, body mass index; PSW, propensity-score weighting; SD, standard deviation.

^a^ Weighted mean or proportion for each group (rounded).

^b^ Rounded.

^c^ Modified Carlson comorbidity score ≥1.

^d^ The exact numbers were not reported because of a Health and Welfare Data Science Center (HWDC) database center policy to avoid numbers in single cells (≤2).

Table S2. Patient characteristics of the study population in the SA-4 [p-stage 3]

|  |  | Patient characteristics before PSW | | |  | Patient characteristics (%) after PSW^a^ | | |
| --- | --- | --- | --- | --- | --- | --- | --- | --- |
|  |  | ACCRT  (n=37) | Observation  (n=10) |  |  | ACCRT | Observation |  |
|  |  | Number (%)^b^ or mean (SD)^b^ | Number (%)^b^ or mean (SD)^b^ | Standardized difference^b^ |  |  |  | Standardized difference^b^ |
| Age (years) |  | 51.76 (10.04) | 61.60 (8.15) | 1.076 |  | 56.24 | 56.24 | ≈ 0 |
| Gender | Female | ^d^ | ^d^ | 0.338 |  | ^d^ | ^d^ | 0.010 |
|  | Male | ^d^ | ^d^ |  |  | ^d^ | ^d^ |  |
| Residency | Non-north | 21 (57) | 6 (60) | 0.066 |  | 61 | 61 | ≈ 0 |
|  | North | 16 (43) | 4 (40) |  |  | 39 | 39 |  |
| Comorbidity | Without | ^d^ | ^d^ | 0.173 |  | ^d^ | ^d^ | ≈ 0 |
|  | With^c^ | ^d^ | ^d^ |  |  | ^d^ | ^d^ |  |
| BMI (kg/m^2^) |  | 22.50 (2.63) | 21.14 (2.77) | 0.504 |  | 20.92 | 20.92 | ≈ 0 |
| Drinking | No | ^d^ | ^d^ | 0.420 |  | ^d^ | ^d^ | 0.013 |
|  | Yes | ^d^ | ^d^ |  |  | ^d^ | ^d^ |  |
| Smoking | No | ^d^ | ^d^ | 0.027 |  | ^d^ | ^d^ | ≈ 0 |
|  | Yes | ^d^ | ^d^ |  |  | ^d^ | ^d^ |  |
| Grade | Poorly | ^d^ | ^d^ | 1.041 |  | ^d^ | ^d^ | 0.025 |
|  | Well/moderately differentiated | ^d^ | ^d^ |  |  | ^d^ | ^d^ |  |
| T-stage | 1–2 | ^d^ | ^d^ | 0.257 |  | ^d^ | ^d^ | ≈ 0 |
|  | 3–4 | ^d^ | ^d^ |  |  | ^d^ | ^d^ |  |
| Tumor location | Upper | ^d^ | ^d^ |  |  | ^d^ | ^d^ |  |
|  | Middle | ^d^ | ^d^ | 0.340 |  | ^d^ | ^d^ | ≈ 0 |
|  | Lower | ^d^ | ^d^ | 0.561 |  | ^d^ | ^d^ | ≈ 0 |
| Tumor size (mm) |  | 45.49 (16.78) | 49.90 (21.02) | 0.232 |  | 50.88 | 50.88 | ≈ 0 |
| Number of lymph node metastases |  | 2.97 (2.69) | 2.80 (2.90) | 0.062 |  | 3.15 | 3.15 | ≈ 0 |

ACCRT, adjuvant concurrent chemoradiotherapy; BMI, body mass index; PSW, propensity-score weighting; SD, standard deviation.

^a^ Weighted mean or proportion for each group (rounded).

^b^ Rounded.

^c^ Modified Carlson comorbidity score ≥1.

^d^ The exact numbers were not reported because of a Health and Welfare Data Science Center (HWDC) database center policy to avoid numbers in single cells (≤2).
